# Long-Term Course of Circulating Elastin, Collagen Type I, and Collagen Type III in Patients with Spontaneous Cervical Artery Dissection: a Prospective Multicenter Study

**DOI:** 10.1007/s12975-023-01207-8

**Published:** 2023-11-10

**Authors:** Silke Zimmermann, Markus Weißenfels, Norma Krümmer, Dominik Michalski, Gesa Weise, Daniela Branzan, Johann Otto Pelz

**Affiliations:** 1 Institute of Laboratory Medicine, Clinical Chemistry and Molecular Diagnostics, University Hospital Leipzig, Leipzig, Germany; 2 Department of Neurology, Heinrich-Braun-Klinikum, Zwickau, Germany; 3 Department of Neurology, Klinikum Altenburger Land, Altenburg, Germany; 4 Department of Neurology, University Hospital Leipzig, Leipzig, Germany; 5 Department of Neurology, Sana Kliniken Leipziger Land, Borna, Germany; 6 Department of Vascular Surgery, University Hospital Leipzig, Leipzig, Germany

**Keywords:** Spontaneous cervical artery dissection (sCAD), Elastin, Collagen, Connective tissue, Vascular extracellular matrix

## Abstract

An impaired integrity of vascular elements and the extracellular matrix (ECM) has been discussed to play a critical role in the pathophysiology of spontaneous cervical artery dissection (sCAD). This study aimed to explore the temporal course of circulating elastin, collagen type I, and collagen type III in patients with sCAD and evaluated their eligibility as diagnostic biomarkers. Patients with sCAD were prospectively enrolled in four German stroke centers. Blood samples were collected at baseline (acute phase), at day 10 ± 3 (subacute phase), and after 6 ± 1 months (chronic phase). Patients with acute ischemic stroke not related to sCAD, healthy probands, and patients undergoing thromboendarterectomy of the carotid artery served as control groups. Serum levels of elastin and collagen types I and III were determined by ELISAs. Fifty-seven patients with sCAD were enrolled. Compared to all three control groups, patients with sCAD had significantly lower levels of elastin and collagen type III at baseline and after 6 months. Compared to healthy probands, patients with sCAD showed similar collagen type I levels at baseline and in the subacute phase, but significantly increased levels after 6 months. As serum levels of elastin, collagen types I and III were not elevated in the acute phase, they do not appear eligible as biomarkers for the diagnosis of sCAD. Persisting low serum levels of elastin and collagen type III towards the chronic phase of sCAD strengthens the hypothesis of a subtle, in most cases clinically inapparent affection of the ECM in patients with sCAD.

## Introduction

Spontaneous cervical artery dissection (sCAD) is a rare vasculopathy with an annual incidence of about 3/100.000 [1, 2]. Despite extensive research, the pathophysiology of sCAD is still not fully understood. Showing a peak in the 5th decade, it is one of the most frequent causes for juvenile stroke [3].

sCAD is characterized by an injury of the vessel wall, leading to an intramural hematoma in the internal carotid artery (ICA) or the vertebral artery (VA). Although there are clinical symptoms like Horner syndrome, unilateral neck pain/headache, and cranial nerve palsy that are indicative for sCAD, the definite diagnosis of sCAD is based on radiological imaging methods and preferably comprises the visualization of the intramural hematoma via magnetic resonance imaging (MRI) [4]. However, the diagnosis of sCAD may be challenging. In the “Cervical Artery Dissection in Stroke Study” (CADISP) trial, about 20% of the initial diagnoses of sCAD had to be revised by the core lab [5].

So far, research for biomarkers in patients with sCAD focused on parameters of inflammation and coagulation [6]. Considering the pathophysiological linkage of sCAD and development of an intramural hematoma, serum biomarkers that are linked to the injury of the vessel wall might be useful in the diagnosis of sCAD. In 2018, Zhu and colleagues found that patients with acute sCAD had higher plasma levels of fibrillin-1 than healthy participants or patients with non-sCAD ischemic stroke [7]. Fibrillin-1 is a component of the extracellular matrix (ECM) and encloses elastin, which is a main component of elastic arteries [8]. Additional components critically impacting the integrity of the vasculature, i.e., the basement membrane, are the family of collagens [9].

We hypothesized, that the injury of the vessel wall in sCAD patients might cause an increase in the serum concentration of vascular and ECM components such as elastin, collagen type I, and collagen type III, and thus explored their long-term course. While comparing serum levels of elastin and collagen types I and III to different control groups, we further examined their eligibility as biomarkers.

## Methods

This multicenter, prospective, non-interventional, explorative study was performed according to the ethical standards laid down in the 1964 Declaration of Helsinki and its later amendment. The study was approved by the local ethics committee of the Medical Faculty at the University of Leipzig (reference number 410/18-ek). All patients or their legal representatives gave their informed and written consent to participate in this study.

### Study Population

Consecutive patients with sCAD were prospectively enrolled in four German stroke centers from May 2018 to June 2023. Besides the existence of typical clinical symptoms, spontaneous cervical artery dissection of the ICA and/or the VA had to be confirmed by radiological imaging, i.e., either by MRI, by computed tomography angiography (CTA), or by digital subtraction angiography (DSA) with the detection of at least one of the following criteria: (1) presence of an intramural hematoma, (2) a long tapering stenosis without signs of atherosclerosis, (3) an intimal flap or a double lumen [4]. Patients with sCAD were further stratified according to the acuity of sCAD: Patients whose first clinical signs of the dissection occurred within 14 days before study enrollment were considered as acute (T0), while those with first symptoms > 14 days before study enrollment were considered as subacute (T1). In addition, in patients with acute sCAD, blood sampling was repeated 10 ± 3 days after study enrollment, i.e., in the subacute phase (T1). Six ± 1 month after the sCAD, all patients were considered as having a chronic sCAD (T3).

Three different populations were selected for comparisons: (1) consecutive patients with an acute non-cervical artery dissection (CAD) ischemic stroke, (2) healthy probands without known cerebro- or cardiovascular disease, (3) patients with an acute ischemic stroke due to a severe (> 70% NASCET), atherosclerotic stenosis of the ipsilateral ICA that underwent thromboendarterectomy.

The demographic characteristics, medical history, clinical presentation, and imaging findings of the patients and the healthy controls were recorded. Patients with sCAD were followed-up at a neurovascular outpatient clinic 6 ± 1 month after study enrollment.

From patients with sCAD, blood samples were collected by venipuncture (serum, S-Monovette®, Sarstedt AG&Co, Germany) within 48 h after hospital admission (at study enrollment), at day 10 ± 3, and at 6 ± 1 months. From patients with non-CAD ischemic stroke, blood samples were collected by venipuncture within 72 h after the onset of the ischemic stroke. Blood samples from patients undergoing thromboendarterectomy were collected by venipuncture before surgery and 7 ± 1 days after surgery. After blood sampling, the tubes were kept in an upright position for 30 min to allow clotting, followed by a centrifugation at 5.500 g for 10 min. The supernatants were then portioned in vials of 400 µl and frozen at − 80 °C until analysis.

### Measurements of Elastin, Collagen Type I, and Collagen Type III

Levels of elastin, collagen type I, and collagen type III in the patients’ sera were determined by enzyme-linked immunosorbent assays (ELISAs) according to the manufacturer’s instructions (collagen type III, elastin: MyBioSource; collagen type I: R&D systems). Samples were diluted according to the test range with either dilution buffer provided by the manufacturer or PBS. The precision of the tests was defined via an intra-assay coefficient of variation (CV): (collagen type I, < 8%; collagen type III, < 8%; elastin, < 9%) and inter-assay (collagen type I, < 10%; collagen type III, < 10%; elastin, < 10%). The test principle was based on sandwich enzyme-linked immune-sorbent assay technology. Capture antibody specific for elastin, collagen type I (the capture antibody for this assay recognizes an epitope in the N-pro peptide (aa 26–161)), and collagen type III was pre-coated onto 96-well plates. Biotin-conjugated antibodies were used as detection antibodies. The standards, test samples and biotin-conjugated detection antibody were added to the wells subsequently and washed with wash buffer. HRP-Streptavidin was added, and unbound conjugates were washed away with a wash buffer. TMB substrates were used to visualize the HRP enzymatic reaction. TMB was catalyzed by HRP to produce a blue color product that changed into yellow after adding an acidic stop solution. The density of yellow is proportional to the target amount of sample captured in plate. After wavelength correction O.D. absorbance values obtained at 450 nm were read, followed by calculation of antigen concentration in each sample by interpolating from the standard curve.

### Statistical Analysis

Participants were stratified as having a spontaneous or traumatic CAD, a non-CAD ischemic stroke, healthy probands, or undergoing thromboendarterectomy due to a symptomatic severe ICA stenosis. Patients with sCAD were further stratified due to the location of the sCAD: ICA or VA.

For statistical calculations, the IBM SPSS Statistics Package Version 29 (IBM Corp., New York, NY, USA) was used. Continuous variables were described by mean ± standard deviation (SD), while categorical variables were expressed as counts with percentages. Extreme outliers were excluded based on Tukey’s hinges (first quartile − 3 * interquartile range (IQR) and third quartile + 3 * IQR), visualized in boxplots [10].

Statistical significance between groups was assessed by chi-square test for categorical variables, by the Mann–Whitney U test for independent, or by Wilcoxon signed-rank test for dependent interval-scaled parameters. Kruskal–Wallis test and Friedman test were used as global non-parametric tests. Spearman signed rank was used for correlation analyses between the onset of first sCAD-related symptoms and the respective serum parameter. A *p* value of < 0.05 was considered statistically significant.

## Results

From May 2018 to June 2023, 72 patients with cervical artery dissection were enrolled. Four (5.6%) patients suffered traumatic CAD due to a minor trauma like choking during judo, chiropractic maneuver, or a punch against the neck. The initial diagnosis of sCAD had to be revised in 7 (9.7%) patients after an extensive diagnostic workup. Four (5.6%) patients with sCAD had their first symptoms > 14 days before study enrollment and were assigned to the subacute (T1) sCAD group. This finally resulted in 57 patients with an acute sCAD. Follow-up data for subacute (T1) and chronic (T2) sCAD patients were available in 38 patients with sCAD. Fifty-four patients with non-CAD ischemic stroke, 11 patients with ischemic stroke and thromboendarterectomy of the ICA, and 80 healthy probands were enrolled for control purpose.

Baseline demographic and clinical data are shown in Table 1. Briefly, patients with sCAD were significantly younger than patients with non-sCAD ischemic stroke and probands. Probands had less cardiovascular risk factors than patients with sCAD and non-CAD ischemic stroke. The mean time from the onset of the first sCAD-related clinical symptoms to study enrollment was 4 days.

**Table 1:** Baseline demographic data of patients with spontaneous cervical artery dissection (CAD) and the control groups. *SD* standard deviation, *NIHSS* National Institute of Health stroke scale. *ICA* internal carotid artery, *VA* vertebral artery

	Patients with acute spontaneous CAD (*n* = 57)	Patients with traumatic CAD (*n* = 4)	Patients with non-CAD ischemic stroke (*n* = 54)	Patients with thromboendarterectomy (*n* = 11)	Probands (*n* = 80)	*p*
Age in years (mean ± SD)	45.7 ± 10.2	54.8 ± 4.3	56.7 ± 13.7	68.2 ± 10.0	57.4 ± 12.9	< 0.001
Female sex (*n*, %)	18 (31.6%)	1 (25%)	15 (27.8%)	2 (18.2%)	56 (70%)	< 0.001
NIHSS at admission (mean ± SD)	2.9 ± 4.2	6.3 ± 7.2	3.8 ± 3.6	1.7 ± 1.1	-	0.037
Time from symptom onset to study enrollment in days (mean ± SD)	4 ± 3	2 ± 1	2 ± 1	9 ± 5	-	< 0.001
Location of CAD (*n*, %)	ICA, 38 (66.7%) VA, 19 (33.3%)	ICA, 3 (75%) VA, 1 (25%)	-	-	-	-
Multiple CAD (*n*, %)	3 (5.3%)	0	-	-	-	-
Arterial hypertension (*n*, %)	29 (50.9%)	3 (75%)	39 (72.2%)	10 (90.9%)	29 (36.6%)	< 0.001
Hyperlipidemia (*n*, %)	29 (50.9%)	0	40 (74.1%)	9 (81.8%)	17 (21.3%)	< 0.001
Diabetes mellitus (*n*, %)	1 (1.8%)	1 (25%)	12 (22.2%)	2 (18.2%)	1 (1.3%)	< 0.001
Current smoking (*n*, %)	20 (35.1%)	0	29 (53.7%)	6 (54.5%)	15 (18.8%)	< 0.001

### Elastin

**Fig. 1:**
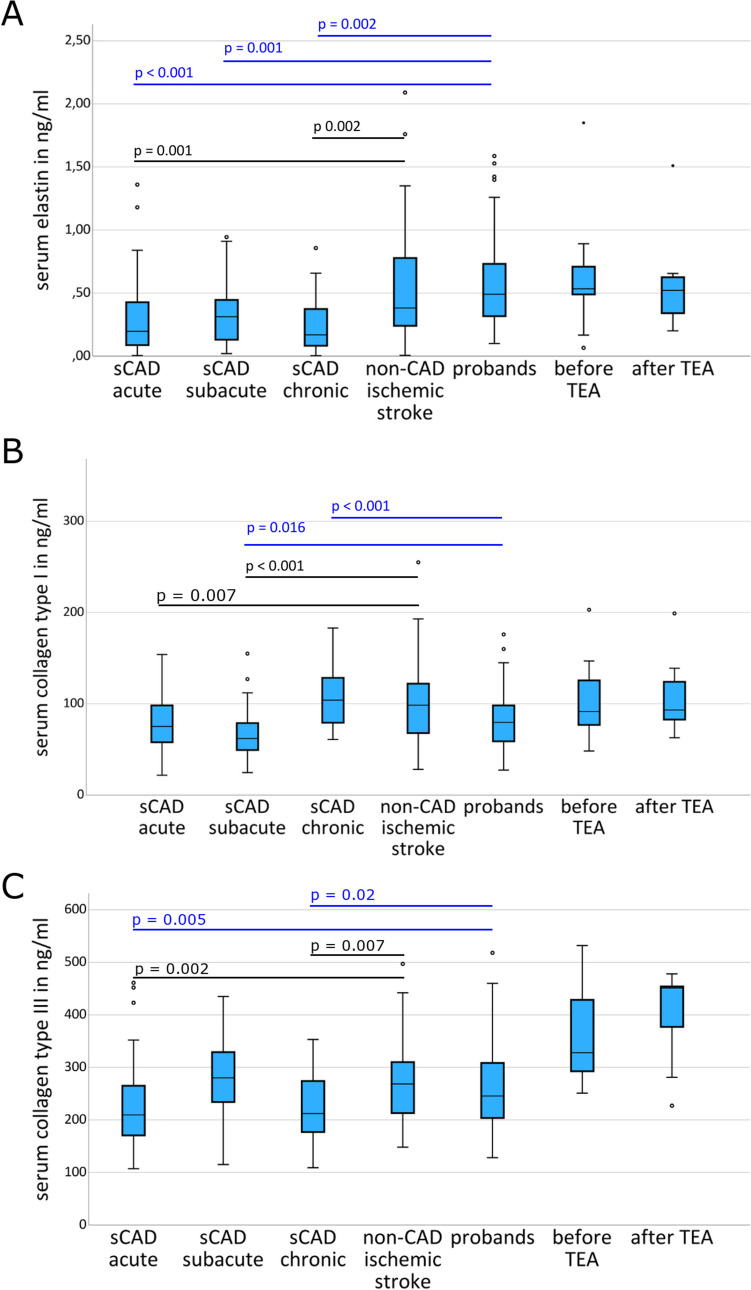
Comparison of serum levels of elastin, collagen type I, and collagen type III between patients with spontaneous cervical artery dissection (sCAD), patients with non-CAD ischemic stroke, probands, and patients with ischemic stroke undergoing thromboendarterectomy (TEA)

**Fig. 2:**
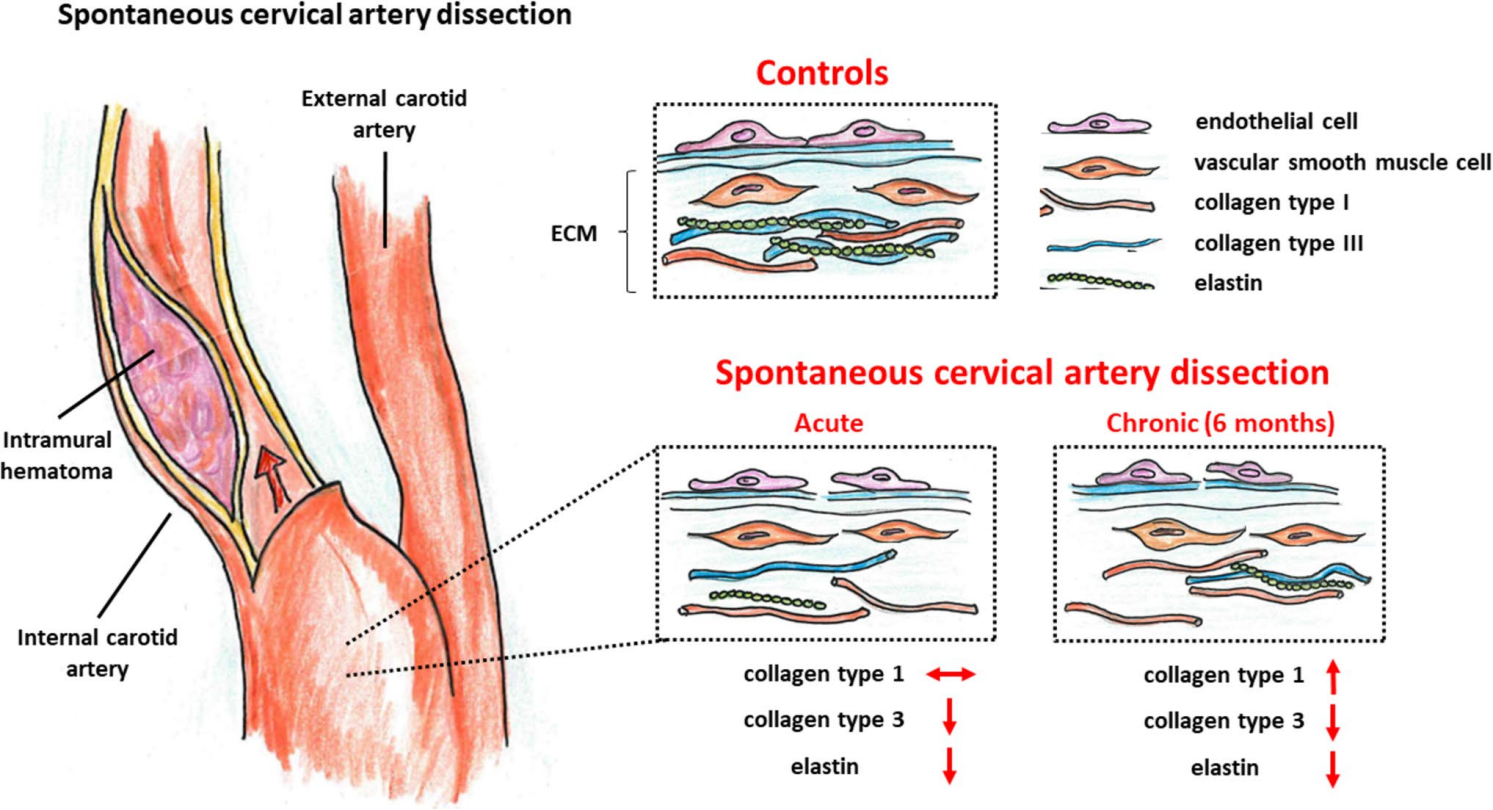
Alterations of components of the vascular extracellular matrix (ECM) in patients with spontaneous cervical artery dissection at baseline (acute phase) and after 6 months (chronic phase) in comparison to controls without known vascular diseases. Red arrows indicate unchanged, lower, or higher levels of indicated components in comparison to healthy controls

**Fig. 3:**
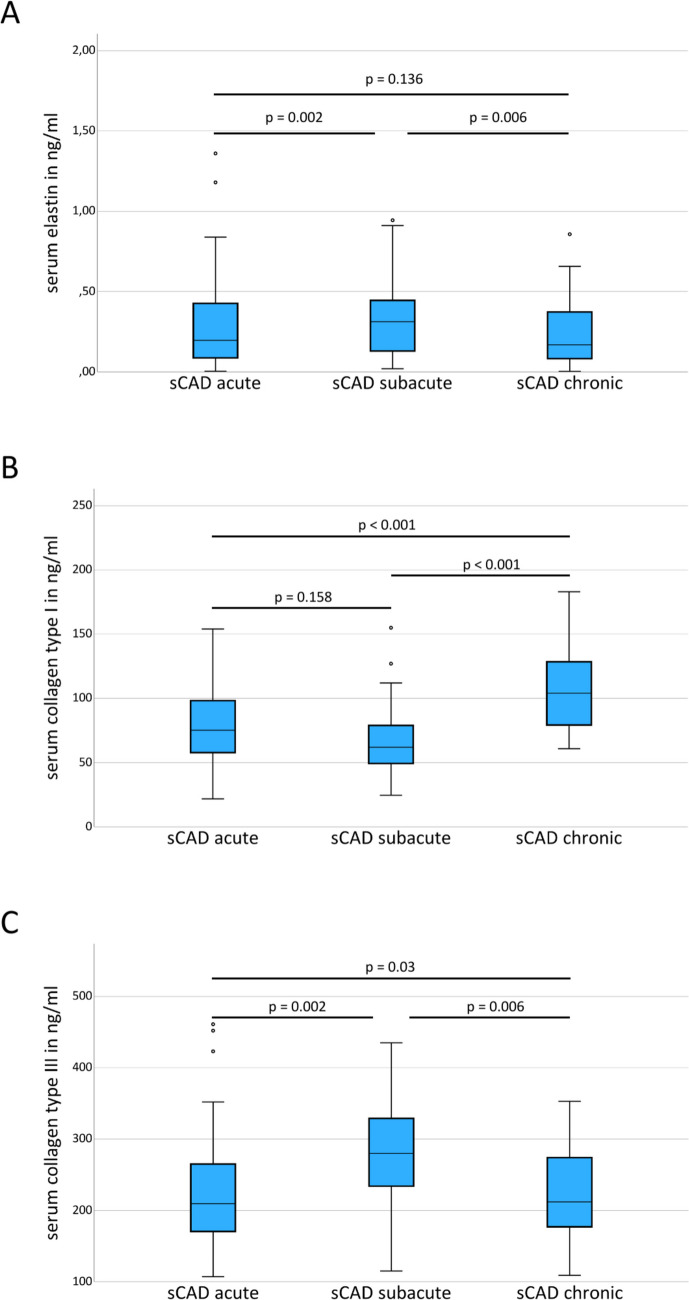
Comparison of serum levels of elastin, collagen type I, and collagen type III between the acute, subacute, and chronic phases in patients with spontaneous cervical artery dissection (sCAD)

Patients with sCAD had lower serum elastin levels in the acute (*p* < 0.001), subacute (*p* = 0.001), and chronic phase (*p* =  < 0.001) compared to probands (Table 2, Figs. 1A, 2). Compared to patients with non-CAD ischemic stroke, serum elastin levels were lower in the acute (*p* = 0.001) and chronic phases (*p* = 0.002), but similar in the subacute phase (*p* = 0.058) of sCAD (Table 2, Fig. 1A). Within the group of patients with sCAD, serum elastin levels were lower at baseline (*p* = 0.002) and after 6 months (*p* = 0.006) compared to the subacute phase (Table 2, Fig. 3A). Serum elastin levels were similar at baseline and after 6 months (*p* = 0.136). Patients with traumatic CAD had similar serum elastin levels like patients with sCAD; however, the small sample size prohibited a statistical comparison. Serum elastin levels at baseline were similar between patients with sCAD of the internal carotid artery and the vertebral artery (0.33 ± 0.34 ng/ml versus 0.19 ± 0.16 ng/ml, *p* = 0.232). There was no correlation between serum elastin levels at baseline and the time from onset of first sCAD-related symptoms to taking the blood sample (*p* = 0.897). Elastin serum levels were similar before and after thrombectomy (*p* = 0.374; Table 2). There were no differences for elastin serum levels between female and male patients with acute sCAD (0.24 ± 0.21 ng/ml versus 0.31 ± 0.33 ng/ml; Mann–Whitney-U, *p* = 0.811), non-CAD ischemic stroke (0.61 ± 0.47 ng/ml versus 0.52 ± 0.47 ng/ml; Mann–Whitney-U, *p* = 0.437), or healthy controls (0.60 ± 0.38 ng/ml versus 0.51 ± 0.33 ng/ml; Mann–Whitney-U, *p* = 0.388).

**Table 2:** Serum levels of components of the extracellular matrix in patients with spontaneous cervical artery dissection (sCAD) and controls. *TEA* thromboendarterectomy

	Patients with sCAD at study enrollment	Patients with sCAD at day 10 ± 3	Patients with sCAD at 6 ± 1 month	Patients with traumatic CAD	Probands	Patients with non-CAD ischemic stroke	Patients before TEA	Patients 7 ± 1 days after TEA
Elastin in ng/ml (mean ± SD)	0.29 ± 0.3	0.35 ± 0.27	0.27 ± 0.23	0.22 ± 0.12	0.57 ± 0.36	0.55 ± 0.46	0.64 ± 0.46	0.56 ± 0.39
Collagen type I in ng/ml (mean ± SD)	79.6 ± 27.6	68.2 ± 26.7	107.7 ± 34.5	-	80.7 ± 30.6	99.5 ± 40.9	103.8 ± 45.0	108.2 ± 42.2
Collagen type III in ng/ml (mean ± SD)	226 ± 78	279 ± 68	224 ± 59	225 ± 49	261 ± 78	267 ± 75	369 ± 97	401 ± 90

### Collagen Type I

Patients with sCAD had similar serum levels of collagen type I at baseline (*p* = 0.906), lower serum levels in the subacute (*p* = 0.016), but higher serum levels of collagen type I in the chronic phase (*p* < 0.001) than probands (Table 2, Figs. 1B, 2). Compared to patients with non-CAD ischemic stroke, serum collagen type I levels were lower at baseline (*p* = 0.007) and in the subacute phase (*p* < 0.001) but similar in the chronic phase (*p* = 0.241; Table 2, Fig. 1B). Within the group of patients with sCAD, serum collagen type I levels were similar between baseline and the subacute phase (*p* = 0.158) and increased after 6 months compared to both, the acute and subacute phases (each *p* < 0.001; Table 2, Fig. 3B). Serum collagen type I levels at baseline were similar between patients with sCAD of the internal carotid artery and the vertebral artery (78.2 ± 25.8 ng/ml versus 82.1 ± 31.3 ng/ml, *p* = 0.887). There was no correlation between serum collagen levels at baseline and time from onset of first sCAD-related symptoms to taking the blood sample (*p* = 0.913). Collagen type I serum levels were similar before and after thrombectomy (*p* = 0.260; Table 2). There were no differences for collagen type I serum levels between female and male patients with acute sCAD (87.0 ± 32.4 mg/ml versus 76.2 ± 24.9 mg/ml; Mann–Whitney-U, *p* = 0.303), non-CAD ischemic stroke (93.6 ± 40.4 ng/ml versus 101.8 ± 41.4 ng/ml; Mann–Whitney-U, *p* = 0.678), or healthy controls (83.1 ± 32.3 ng/ml versus 75.1 ± 26.3 ng/ml; Mann–Whitney-U, *p* = 0.449).

### Collagen Type III

Compared to healthy controls and patients with non-CAD ischemic stroke, patients with sCAD had lower serum levels of collagen type III at baseline (*p* = 0.005, *p* = 0.002) and 6 months later in the chronic phase (*p* = 0.02, *p* = 0.007). In the subacute phase, no difference was found for the serum levels of collagen type III between patients with sCAD, patients with non-CAD ischemic stroke, and probands (Table 2, Figs. 1C, 2). Within the group of patients with sCAD, serum levels of collagen type III were lower at baseline (*p* = 0.002) and after 6 months (*p* = 0.006) compared to the subacute phase (Table 2, Fig. 3C). Serum levels of collagen type III were higher at baseline than after 6 months (*p* = 0.03; Table 2, Fig. 3C). Patients with traumatic CAD had similar serum levels of collagen type III like patients with sCAD; however, small sample size prohibited a statistical comparison. Serum levels of collagen type III at baseline were similar between patients with sCAD of the internal carotid artery and the vertebral artery (234 ± 85 ng/ml versus 211 ± 62 ng/ml, *p* = 0.411). There was no correlation between serum levels of collagen type III at baseline and time from onset of first sCAD-related symptoms to taking the blood sample (*p* = 0.168). Collagen type III serum levels were similar before and after thrombectomy (*p* = 0.214; Table 2). There were no differences for collagen type III serum levels between female and male patients with acute sCAD (214 ± 64 versus 232 ± 84 mg/ml; Mann–Whitney-U, *p* = 0.719), non-CAD ischemic stroke (243 ± 70 versus 276 ± 76 ng/ml; Mann–Whitney-U, *p* = 0.145), or healthy controls (262 ± 71 versus 260 ± 94 ng/ml; Mann–Whitney-U, *p* = 0.713).

## Discussion

One main finding of this prospective and longitudinal multicenter study was that neither elastin, collagen type I, nor collagen type III serum levels were elevated in the acute phase of sCAD compared to both, healthy probands and patients with non-CAD ischemic stroke. Secondly, in comparison to the control groups, elastin and collagen type III serum levels were even significantly lower at baseline *and* after 6 months, while collagen type I serum levels showed a significant increase after 6 months. Thus, elastin, collagen type I, and collagen type III serum levels do not appear eligible as serum biomarkers for the initial diagnosis of sCAD. It is even questionable whether a rather circumscribed vascular injury like in sCAD can cause a relevant increase in the serum levels of vascular and ECM proteins even days after the index event. Hence, we also did not find a difference in serum levels of elastin, collagen type I, and collagen type III before and after thromboendarterectomy of the ICA, in which a significant affection of the vascular wall unequivocally happened during the surgical procedure.

Although the initial hypothesis could not be confirmed, this study provides valuable insights into the pathogenesis of sCAD. Thereby, low serum levels of elastin and collagen type III at baseline and after 6 months support the hypothesis of an underlying, in most cases subtle and clinically inapparent alteration of vascular and ECM components in patients with sCAD [11]. Brandt and colleagues demonstrated that in semi-quantitative analyses, more than half of the patients with sCAD showed ultrastructural aberrations in skin biopsies, which comprised elastic fiber abnormalities with mini-calcifications and fragmentation, and composite fibrils within collagen bundles that in some patients resembled the aberrations found in Ehlers–Danlos syndrome types II or III [11]. The mean diameter of collagen fibrils in the skin was lower, while fibril density was higher in patients with sCAD compared to controls [13]. Collagen type I and collagen type III were present in normal as well as abnormal sections of collagen fibrils. A strong increase of both collagens was detected in abnormal sections, which points to a dysbalance of the collagen subtypes in the composition of the dermal connective tissue in sCAD patients [14]. Moreover, the quantitative analysis of collagens in dermal fibroblast cultures of sCAD patients showed a lower ratio of collagen type III/collagen type I in patients with sCAD compared to controls [15]. As demonstrated in our study, such a reduced ratio of types III/I collagen could be due to a decrease in collagen type III in patients with sCAD and/or an increase of collagen type I in patients with non-CAD ischemic stroke, that served as controls. However, some patients with sCAD only showed aberrations of elastic fibers (with fragmentation and mini-calcifications) but no significant alterations in the morphology of collagen fibrils [16]. These findings of aberrations of the dermal connective tissue could be reliably replicated in the meantime [14, 16–19]. Since most skin biopsies were performed once with a close temporal relationship to the diagnosis of sCAD, the significance of these findings is limited with respect to chronic alterations. In a small group of eight patients with sCAD, a second skin biopsy 9 to 23 months after the first biopsy showed identical ultrastructural findings and was ranked at the same level of pathomorphological severity in both analyses [12]. Noteworthy, in most patients with sCAD, a family history of symptomatic sCAD was rare and, despite anecdotal reports of monogenetic pathologies in collagen and elastin genes [20, 21], inherited connective tissue disorders seemed exceptional [22]. This raises the hypothesis that in sCAD the vascular and associated ECM proteins themselves are intact but that rather the quantity of their synthesis and/or the assembly of more complex fibrils is affected. This hypothesis is supported by recent findings, again in skin biopsies, that patients with recurrent sCAD showed a significantly different expression of 25 proteins of whom 13 played a role in the structural integrity of the connective tissue. Notably, these proteins showed clustering to a collagen/elastin cluster with a reduced abundance of elastin, collagen type IV, collagen type XXIII, and collagen type I, while the expression of collagen type XII was increased [23].

So far, in the absence of taking biopsies from the (dissected) cervical artery itself, skin biopsies served as a surrogate to assess connective tissue aberrations in patients with sCAD. Hence, the connective tissue of the skin was considered to be a “window to the arteries” [17]. As demonstrated in this study, measuring serum levels of the main components of the vascular and the associated ECM fitted well to these known ultrastructural aberrations of the skin. From the clinician’s perspective, measuring serum proteins is more feasible than the semi-quantitative rating of skin biopsies which requires a high level of expertise during the whole process of taking, preparing, and analyzing the specimens.

An altered composition of the vasculature and the associated ECM would also affect the vessel’s elastic properties. Mice with just one elastin allele (Eln^+/−^) had reduced elastin levels (50–60% of normal elastin levels) but showed a similar circumferential stress-stretch behavior like elastin wildtype mice. However, a further reduction of elastin levels to 30–40% of normal in hBAC-mNull mice was associated with an incomplete remodeling and impaired adaption to pressure [8]. Abnormal elastic properties with higher stiffness of the non-dissected carotid and vertebral artery but not of the brachial artery were also reported in sCAD patients [24–26]. Moreover, in patients with sCAD, signs of tissue weakening along the tunica media/tunica adventitia were also found in biopsies of the superficial temporal artery, which was suggestive of a generalized arteriopathy [27].

The transient increase of elastin and collagen type III serum levels in the subacute phase, and of collagen type I in the chronic phase of sCAD might be due to a remodeling of the vascular and ECM components. This vascular remodeling may be linked to the resolution of the intramural hematoma and alters the composition of the vasculature and the associated ECM, resulting in a persistent increase of collagen type I. A decrease in the degree of stenosis and recanalization of the afore-occluded artery was observed in more than half of all sCAD patients [28].

Despite its strength of being a prospective and multicenter study, this study also has some limitations. First, our findings warrant confirmation in a larger cohort of patients with sCAD. Secondly, it is not clear, whether low serum levels of vascular and ECM proteins are associated with morphological alterations of the carotid or vertebral artery. However, in the few histopathological examinations of dissected carotid arteries, an irregular and disorganized arrangement of elastin in the tunica media but also a rarefication of elastic material was described [17, 29]. Moreover, correlation between the arterial vessel wall and the dermal connective tissue could be performed in some patients and showed much more pronounced findings in the carotid artery, particularly involving elastic fibers [17].

Moreover, there were only four patients with traumatic CAD due to a minor trauma in this study. Thus, statistical analyses could not be performed. Noteworthy, serum levels for elastin and collagen type III in patients with traumatic CAD were nearly identical to patients with acute sCAD. Since minor neck traumas occur regularly but rarely cause traumatic CAD, an underlying aberration of the connective tissue could also be a risk factor for traumatic CAD.

This study shows that patients with sCAD are characterized by persisting low serum levels of elastin and collagen type III, while, probably because of vascular remodeling, collagen type I levels increased strongly towards 6 months. This observation strengthens the hypothesis of a subtle, in most cases clinically inapparent affection of the vasculature and the associated ECM in patients with sCAD [11]. These findings might also have implications for other vascular diseases like intracranial aneurysms or acute dissection of the aorta, whose pathogenesis is currently also not fully understood [30, 31].

## Data Availability

The dataset underlying this study is available from the corresponding author on reasonable request for any qualified investigator.

## Declarations

### Full Data Access and Statements

Johann Otto Pelz as the Principal Author has full access to the data used in the analyses in the manuscript, and takes full responsibility for the data, the analyses and interpretation, and the conduct of the research.
